# Ozone Exposure and Gestational Diabetes in Twin Pregnancies: Exploring Critical Windows and Synergistic Risks

**DOI:** 10.3390/toxics13020117

**Published:** 2025-02-01

**Authors:** Anda Zhao, Yuanqing Xia, Ruoyu Lu, Wenhui Kang, Lili Huang, Renyi Hua, Shuping Lyu, Yan Zhao, Jianyu Chen, Yanlin Wang, Shenghui Li

**Affiliations:** 1 School of Public Health, Shanghai Jiao Tong University School of Medicine, Shanghai 200025, China; zad1002@163.com (A.Z.); luruoyu0702@163.com (R.L.); 18717805230@163.com (W.K.); lili_huang@shsmu.edu.cn (L.H.); 2 Huadong Hospital, Fudan University, Shanghai 200040, China; 3 Hainan Branch, Shanghai Children’s Medical Center, School of Medicine, Shanghai Jiao Tong University, Sanya 572000, China; 4 School of Public Health, Li Ka Shing Faculty of Medicine, The University of Hong Kong, Hong Kong, China; 13162575998@163.com; 5 International Peace Maternity and Child Health Hospital, Shanghai Jiao Tong University School of Medicine, Shanghai 200030, China; renyi3551_cn@163.com (R.H.); lv0217shuping@163.com (S.L.); 6 Shanghai Key Laboratory of Embryo Original Diseases, Shanghai 200030, China; 7 Shanghai Municipal Key Clinical Specialty, Shanghai 200030, China; 8 The People’s Hospital of Nujiang Lisu Autonomous Prefecture, Lushui 673199, China; yanzhao20241221@126.com; 9 College of Public Health, Shanghai University of Medicine & Health Sciences, Shanghai 201318, China; chenjy22@sumhs.edu.cn; 10 MOE-Shanghai Key Laboratory of Children’s Environmental Health, Shanghai Jiao Tong University School of Medicine, Shanghai 200092, China

**Keywords:** ozone, gestational diabetes mellitus, twin pregnancies, distributed lag non-linear models, critical windows

## Abstract

The relationship between ozone (O_3_) exposure and gestational diabetes mellitus (GDM) in twin pregnancies remains unexplored. This study aimed to investigate the association between O_3_ exposure and GDM risk in twin pregnancies, and to explore the synergistic effects of O_3_ exposure with other maternal factors. A total of 428 pregnancies recruited from a prospective twin cohort were included. Cox proportional hazard models with distributed lag non-linear models (DLNMs) were applied to examine the associations between O_3_ exposure and the risk of GDM and to identify the critical windows. The multiplicative and additive interaction were further analyzed to test the synergistic effects. A 10 μg/m^3^ increase in average O_3_ exposure during the 12 weeks before pregnancy was associated with a 26% higher risk of GDM. The critical windows were identified in the period from the 3rd week before gestation to the 2nd gestational week as well as from the 17th to 19th gestational week. There were synergistic effects between high O_3_ exposure during preconception and advanced maternal age, and a history of preterm birth/abortion/stillbirth. Periconceptional O_3_ exposure could increase the risk of GDM in twin pregnancy women, and the synergism of O_3_ exposure with certain GDM risk factors was observed.

## 1. Introduction

Gestational diabetes mellitus (GDM) is defined as diabetes in the pregnant women who did not have overt diabetes prior to gestation [1]. The global prevalence of GDM varies widely, ranging from 1.0% to over 30.0% [2,3]. GDM has been shown to have both short- and long-term negative health impacts on mothers as well as their children. The short-term consequences include increased risks of gestational hypertensive disease, stillbirth, preterm birth, cesarean sections, and the occurrence of macrosomia at delivery [1,4,5,6]. For long-term outcomes, mothers with GDM are at an elevated risk for developing type 2 diabetes, ischemic heart disease, nonalcoholic fatty liver diseases and hypertension, and their offsprings face a greater susceptibility to impaired cognitive ability, cardiovascular disease, and metabolic complications later in life [1,6,7].

There is growing evidence linking atmospheric pollution exposure to GDM, and the association between ozone (O_3_) and GDM is particularly drawing attention in recent years [8,9,10,11,12]. O_3_ pollution has been considered as an important global health threat since ongoing climate change and anthropogenic emissions are causing the rising of ground-level O_3_ concentrations in majority of areas and countries all around the world [10]. Several animal experiments confirmed that exposure to O_3_ can disrupt glucose homeostasis [13,14]. Recent epidemiological studies have also established associations between O_3_ exposure and the risk of GDM [11,15,16,17,18]. However, it is worth noting that all the existing data were limited to singleton pregnancies, and there is a lack of research with focus on the association between O_3_ exposure and the development of GDM in twin pregnancies.

The incidence of twin pregnancies has continued to rise over the past two decades due to delayed childbearing and the wide use of assisted reproductive technologies (ART) [19]. The proportion of twin births in China has exceeded 3.0% [20]. It has been indicated that twin pregnancy is an independent risk factor for the development of GDM [21,22,23]. Moreover, GDM in twin pregnancies might exacerbate adverse health outcomes, including preeclampsia, perinatal mortality, and induction rates [24,25]. Therefore, it is worth investigating the relationship between O_3_ exposure and the occurrence of GDM in twin pregnancies.

In addition, it is crucial to highlight that very few studies have explored whether there is an interaction between O_3_ exposure and the individual characteristics of pregnant women in the development of GDM. A recent study has shown an interaction of seasonal variation and pre-pregnancy body mass index (BMI) in the risk of GDM [26].

The aim of the present study was to examine the association of pre-conceptional and prenatal exposure to O_3_ with the risk of GDM in twin pregnancies, and to explore the synergistic effect between O_3_ and maternal characteristics on GDM development.

## 2. Methods

### 2.1. Original Cohort

This research is part of the ongoing Shanghai Twin Pregnancy Birth Cohort (STPBC) Study, a prospective birth cohort designed to investigate the effects of environmental and behavioral exposures during the periconception period on the outcomes of twin pregnancies. This study is conducted at the International Peace Maternity and Child Health Hospital, affiliated with Shanghai Jiao Tong University School of Medicine. Beginning 15 November 2018, all women with twin pregnancies in early gestation (less than 14 weeks) were invited to participate in the STPBC study during their initial visit to the twin clinic with participation limited to those who provided informed consent. Further details on the STPBC study have been published in our previous research [27]. The STPBC Study was approved by the ethics committee of Shanghai Jiao Tong University School of Medicine (SJUPN-201717).

### 2.2. Study Population

Between November 2018 and December 2023, a total of 675 twin pregnant women were enrolled in the present study. However, 247 participants were excluded due to factors such as preexisting maternal diseases, fetal chromosomal abnormalities, severe structural defects, spontaneous/iatrogenic reduction, abortion, stillbirth, or missing data on home address or GDM status. As a result, 428 women with twin pregnancies were included in the final analysis (Figure S1).

### 2.3. Assessment of Ambient Pollutant Exposure, Temperature, and Relative Humidity

The residential addresses of the pregnant women were collected at their initial antenatal examination. Each residential address was converted into longitude and latitude coordinates to determine the concentration of O_3_ and PM_2.5_ in the center of the nearest grid area. Data for a daily maximum 8 h O_3_ and PM_2.5_ during the study period were obtained from Tracking Air Pollution in the China database (http://tapdata.org.cn, accessed on 1 May 2024) and the spatial resolution of both O_3_ and PM_2.5_ was 10 km [28,29,30,31,32,33]. Figure S2 shows the monthly fluctuation in O_3_ concentration from January 2018 to December 2023. The weekly O_3_ and PM_2.5_ exposure from the 12th week before gestation to the 35th gestational week were calculated, considering the 12 weeks before pregnancy, is a plausible biologically relevant window of O_3_ exposure that might influence menstrual cycles and ovulation prior to gestation [34,35]. The details on the calculation of O_3_ and PM_2.5_ measurements have been reported previously [28,29,30,31,32,33]. Average values for O_3_ and PM_2.5_ in both the week and the trimester scale were utilized to assess atmospheric exposure in our study. Average daily values for other ambient pollutants (PM_10_, SO_2_, NO_2_, and CO) were calculated by applying the inverse distance weighted (IDW) method with data being obtained from air quality monitoring stations in Shanghai. Average daily values for temperature and relative humidity were calculated based on the historical reanalysis datasets from the National Aeronautics and Space Administration (NASA) (www.xihe-energy.com, accessed on 1 May 2024).

Among the latest documents released by the World Health Organization (WHO), the Global Air Quality Guidelines (AOG 2021) explicitly define the threshold for ozone; however, only for the general population [36]. According to the WHO AQG 2021 [36], daily maximum 8 h O_3_ concentration limits are 100 µg/m^3^. Considering that the specific O_3_ exposure thresholds exclusively for pregnancy were unavailable, we used the general WHO guideline thresholds as a reference to define high and low O_3_ concentration cases in our analysis. Accordingly, average O_3_ exposure was categorized into high exposure (≥100 µg/m^3^) and low exposure (<100 µg/m^3^).

### 2.4. Outcome Measurements

All participants were required to complete GDM screening utilizing a 2 h 75 g oral glucose tolerance test (OGTT) between the 24th and 28th weeks of pregnancy and undergo fasting plasma glucose (FPG) from the 32nd to 34th gestational weeks. GDM was diagnosed based on the recommendations of the International Association of Diabetes and Pregnancy Study Groups (IADPSG): FPG ≥ 5.1 mmol/L (≥92 mg/dL), 1 h plasma glucose ≥ 10.0 mmol/L (≥180 mg/dL), or 2 h plasma glucose ≥ 8.5 mmol/L (≥153 mg/dL) [37].

### 2.5. Covariates

Covariates included maternal age, ethnicity (Han, other), education level (junior school or below, high school, college or graduate school or above), family monthly income per capita (RMB <5000, RMB 5000–7999, RMB ≥8000), first gestation (yes, no), primipara (yes, no), history of abortion/preterm birth/still birth (yes, no), chorionicity [monochorionic monoamniotic (MCMA)/monochorionic diamniotic (MCDA), dichorionic diamniotic (DCDA)], pregnancy via ART (yes, no), preconception tobacco/alcohol use (yes, no), pre-pregnancy BMI, anemia (yes, no), gestational hypertension (yes, no), and thyroid disease (yes, no). Maternal age was further categorized into <35 years and ≥35 years based on the widely recognized clinical classification that women aged 35 years or older is defined as advanced maternal age, a threshold associated with increased risks for pregnancy-related outcomes [38].

### 2.6. Statistical Analyses

Statistical descriptions were provided using percentages for categorical variables, and group differences were examined through the Chi-squared test.

The Cox proportional hazard models were applied to evaluate the association between average O_3_ exposure during different gestational periods and GDM. We fitted the following four models. In the crude model, only average O_3_ exposure was included. Model I was adjusted for temperature and relative humidity. Model II was further adjusted for sociodemographic characteristics (advanced maternal age, ethnicity, education level, and family income), history of preterm birth/abortion/still birth, first gestation, primipara, chorionicity, and pregnancy via ART in addition to the factors included in model I. Model III, based on the covariates in Model II, pregnancy health indicators, including maternal tobacco/alcohol use, pre-pregnancy BMI, anemia, thyroid disease, and gestational hypertension were simultaneously adjusted.

Double-pollution models were utilized to examine the potential confounding effects, in which one of the air pollutants, including PM_2.5_, PM_10_, SO_2_, NO_2_, and CO, was applied to adjust the model [8].

A distributed lag non-linear model (DLNM) incorporated in Cox proportional hazard models was then utilized to identify the susceptible weeks of O_3_ exposure for the development of GDM. In this model, a cross-basis matrix for O_3_ was created, with the exposure-response dimension being set as linear by default, and the lag-response dimension defined by natural cubic splines. Based on Akaike information criterion (AIC), the degree of freedom for the optimal model was set as 5. The ambient temperature and relative humidity were included in the models using natural cubic splines, with the degrees of freedom for both being set as 2 in the exposure-response and the lag-response dimension. In addition, the multiplicative and additive interaction between high O_3_ exposure (≥100 μg/m^3^) during different periods and maternal GDM risk factors (age and history of preterm birth/abortion/still birth) on GDM was tested. Relative excess risk due to interaction (RERI) was utilized to determine the existence of additive interaction, with RERI > 0 indicating a statistically significant additive interaction. Subsequently, the stratification analyses were performed according to maternal characteristics.

We also conducted sensitivity analyses to test the robustness of our results. First, we used natural splines with 6 or 7 degrees of freedom for the O_3_ lag constraint, and with 3 degrees for the temperature or relative humidity non-linear constraint in the cross matrix to assess the hazards of GDM separately. Second, we restricted the study population to those subjects with Han ethnicity, without DCDA, without tobacco/alcohol use, or without pre-pregnancy obesity.

All statistical analyses were performed using R software version 4.3.1 (R Foundation for Statistical Computing, Vienna, Austria). A two-sided *p* value < 0.05 was considered as significant difference.

## 3. Results

### 3.1. Baseline Characteristics of Participants

Table 1 summarizes the baseline characteristics of participants. Of the 428 twin pregnant women, 72 (16.82%) were diagnosed with GDM. Participants with GDM tended to have a higher age and pre-pregnancy BMI (*p* < 0.05). Levels of O_3_ and other air pollutant exposure during different periods among participants were presented in Table S1. We also explored the distribution of maternal age, history of abortion/preterm birth/still birth, and pre-pregnancy obesity between the two groups of high vs. low O_3_ exposure before pre-pregnancy. We found that there were no significant differences between the two groups (Table S2) as well. The median average O_3_ exposure from pre-pregnancy to the second trimester was consistently above 100 μg/m^3^.

### 3.2. Associations Between Average O_3_ Exposure During Different Periods and the Risks of GDM

Table 2 shows the impact of average O_3_ exposure during different periods on GDM. After adjusting for covariates, it was found that there was a significant association between average O_3_ exposure during the 12 weeks before pregnancy and the incidence of GDM; a per 10 μg/m^3^ increase in O_3_ was associated with a 26% (95% CI: 7–48%) higher risk of GDM (*p* < 0.05). In addition, it was also indicated that participants with high exposure to O_3_ (≥100 μg/m^3^) during the preconception period were more likely to develop GDM (HR: 2.89, 95% CI: 1.36–6.12). By contrast, these associations were not observed in the first and the second trimester. Consistent changes were found in the double-pollution models. Moreover, we also explored the association between O_3_ exposure for each month before pregnancy and GDM (Figure S3). Similar results were observed in general.

### 3.3. Critical Windows of Weekly O_3_ Exposure for GDM Risks

**Figure 1:**
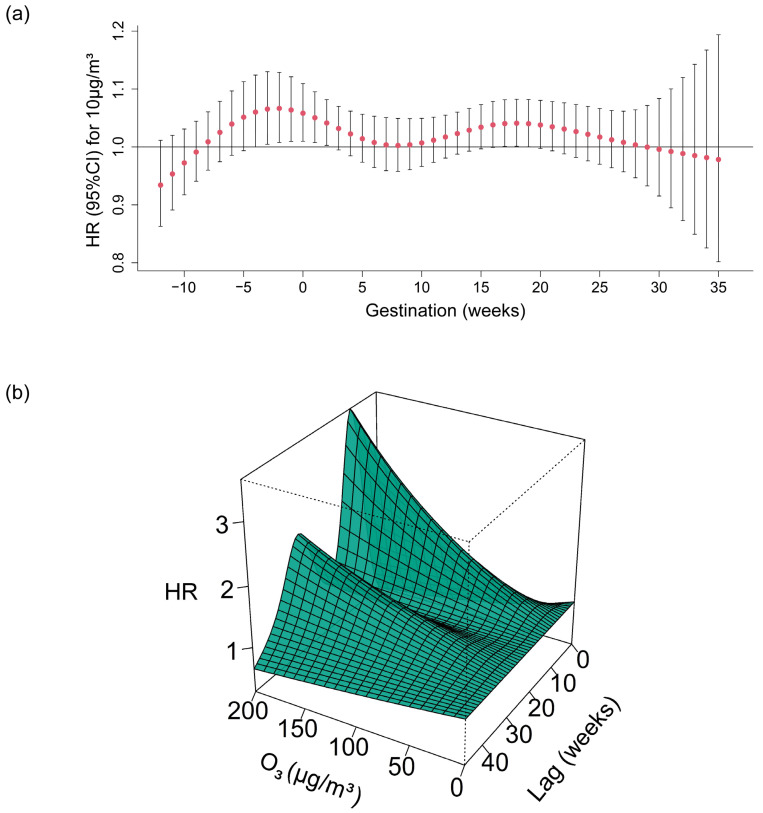
Weekly associations of O_3_ exposure (per 10 μg/m^3^) with the development of GDM. (**a**) and (**b**) showed the weekly association between O_3_ and GDM of pregnancy in 2D and 3D plots, respectively. Adjusted for temperature and relative humidity, sociodemographic characteristics (advanced maternal age, ethnicity, education level, and family income), history of preterm birth/abortion/still birth, first gestation, primipara, chorionicity, pregnancy via assisted reproductive technology, pregnancy health indicators, including maternal tobacco/alcohol use, pre-pregnancy body mass index, anemia, thyroid disease, and gestational hypertension. O_3_: ozone; GDM: gestational diabetes mellitus; HR: hazard ratio; CI: confidence interval.

Figure 1 shows the association between O_3_ exposure and GDM in 2D and 3D plots. The hazard of GDM was associated with O_3_ exposure from the 3rd week before gestation to the 2nd gestational week and from the 17th to the 19th gestational week. Figure 1a indicates that the strongest effect of O_3_ exposure was observed in the 2nd week before gestation (HR: 1.07, 95% CI: 1.08–1.13). Figure 1b depicts the effects of O_3_ exposure on GDM with different lag weeks, with two significant peaks by lag week from lag 9 weeks to lag 14 weeks and from lag 29 weeks to lag 31 weeks.

### 3.4. Interaction Between High O_3_ Exposure During Different Periods and Maternal Characteristics on GDM Risks

**Figure 2:**
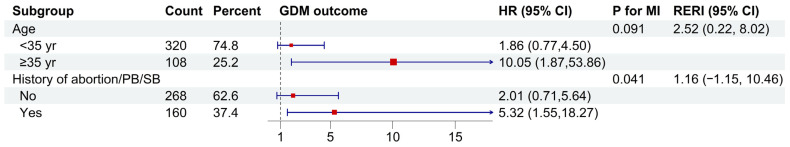
Interaction between high O_3_ exposure during the 12 weeks before pregnancy and maternal characteristics on GDM risks. Adjusted for temperature and relative humidity, sociodemographic characteristics (advanced maternal age, ethnicity, education level, and family income), history of preterm birth/abortion/still birth, first gestation, primipara, chorionicity, pregnancy via assisted reproductive technology, pregnancy health indicators, including maternal tobacco/alcohol use, pre-pregnancy body mass index, anemia, thyroid disease, and gestational hypertension. O_3_: ozone; GDM: gestational diabetes mellitus; HR: hazard ratio; CI: confidence interval; MI: multiplicative interaction; RERI: relative excess risk due to interaction.

The results presented in Figure 2 indicate a significant additive interaction between high O_3_ exposure (≥100 μg/m^3^) during the preconception period and an advanced maternal age in relation to GDM (RERI: 2.52, 95% CI: 0.22–8.02). Additionally, a significant multiplicative interaction was observed between high O_3_ exposure during the preconception period and a history of abortion, preterm birth, or stillbirth (*p* for multiplicative interaction < 0.05). However, these associations were not observed in the first and the second trimester (Table S3).

### 3.5. Stratification Analyses

**Figure 3:**
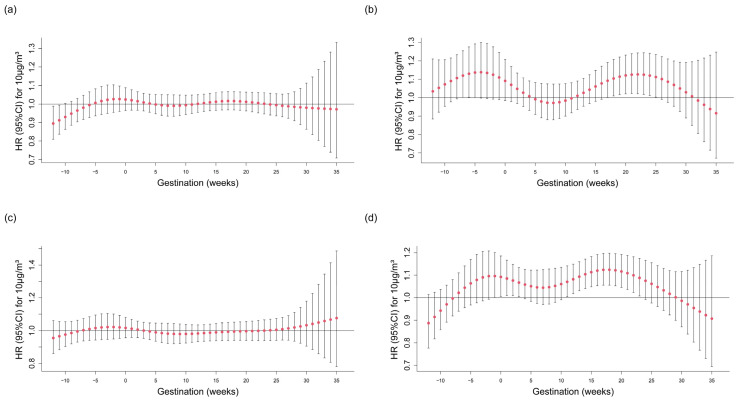
Stratification analyses: weekly associations between high O_3_ exposure during the 12 weeks before pregnancy and GDM for different maternal characteristics. (**a**,**b**) showed the association in women without and with advanced maternal age, respectively. (**c**,**d**) showed the association in women without and with history of preterm birth/abortion/still birth, respectively. Adjusted for temperature and relative humidity, sociodemographic characteristics (advanced maternal age, ethnicity, education level, and family income), history of preterm birth/abortion/still birth, first gestation, primipara, chorionicity, pregnancy via assisted reproductive technology, pregnancy health indicators, including maternal tobacco/alcohol use, pre-pregnancy body mass index, anemia, thyroid disease, and gestational hypertension. O_3_: ozone; GDM: gestational diabetes mellitus; HR: hazard ratio; CI: confidence interval.

In the stratification analyses, we estimated the effects of weekly O_3_ exposure on GDM according to maternal age and history of abortion/preterm birth/stillbirth. The similar findings were generally shown that women with advanced age, and history of abortion, preterm birth, or stillbirth were more sensitive to O_3_ exposure across the preconception period and the second trimester (Figure 3).

### 3.6. Sensitivity Analyses

Utilizing natural splines with 6 and 7 degrees of freedom for the O_3_ lag constraint and with 3 degrees for the temperature or relative humidity non-linear constraint, we still observed the similar results that the significant lag windows for O_3_ spanned the preconception period and the first trimester (Figure S4). After excluding participants of minority ethnicities, carrying DCDA, using tobacco/alcohol, or pre-pregnancy obesity, we found that the average O_3_ exposure during the preconception period remained positively associated with GDM (Table S4) and the critical windows were consistent with primary analyses (Figure S4).

## 4. Discussion

Based on the STPBC Study, for the first time we explored the association between O_3_ exposure during different periconception periods and the risk of GDM among twin pregnant women. Our results suggested that there was a significant association between higher O_3_ exposure during pre-pregnancy and an increased incidence of GDM, and the periods of 3rd week before gestation to the 2nd gestational week and the 17th to the 19th gestational week might be the potential critical windows. More importantly, the synergistic effects were observed between O_3_ exposure and advanced maternal age and having history of abortion/preterm birth/stillbirth on the risk of GDM.

The incidence of twin pregnancies in recent years is an increasing trend [20] and several studies reported that twin pregnancies were associated with an elevated risk of GDM, compared with singleton pregnancies [39,40]. Another cohort study conducted in Shanghai reported that the incidence of GDM in singleton pregnancies was 12.32% [20]. In contrast, our study found a substantially higher GDM incidence of 16.82% in twin pregnancies.

In recent years, a growing number of epidemiological studies have been conducted to investigate the association in singleton pregnancies [11,15,16,17,18]. The results of these studies consistently showed that O_3_ exposure during the conception period increased the risk of GDM [11,15,16,17,18]. Nevertheless, so far there is a research gap in twin pregnancies. This is the first study focusing on twin pregnancies, observing that there was a significant positive association between average O_3_ exposure during the 12 weeks before pregnancy and the development of GDM. To our knowledge, to date very few studies have explored the risk of O_3_ exposure during pre-pregnancy on GDM in singleton pregnancies. The two studies from the US, in which O_3_ concentrations ranged from 29.71 to 41.3 ppb during pre-pregnancy, found a significantly inverse association between O_3_ exposure and GDM [41,42]. By contrast, a study in Taiwan, O_3_ concentrations being around 27.5 ppb, suggested a positive association, which supported our findings to a large extent; however, the association did not reach the significant level on statistics [43]. A more recent study conducted in Beijing also indicated that the elevated risk of GDM is associated with the increased O_3_ exposure during the first month before pregnancy [16]. Based on the existing data, the contradictory results might be due to the ethnic differences and the physiological variations between twin and singleton pregnancies. One of the primary differences between twin pregnancies and singleton pregnancies is the increased placental mass in twin pregnancies, which produces higher levels of hormones such as human placental lactogen and progesterone [21,22,23]. These hormones can interfere with insulin sensitivity, leading to insulin resistance and GDM [21,22,23]. Furthermore, the increased levels of oxidative stress biomarkers including malondialdehyde (MDA) and decreased antioxidant ability were found in twin pregnancy women, indicating twin pregnancies could induce higher level of oxidative stress [44]. Oxidative stress can damage the pancreatic β-cells, resulting in decreased insulin secretion and interfere with the normal function of insulin receptors, leading to insulin resistance, and thereby increasing the risk of GDM [45]. In addition, O_3_ exposure could exert oxidative stress by producing 4-hydroxynonenal (4HNE) and decreasing superoxide dismutase (SOD), increase circulating metabolites of glycolysis and promote gluconeogenesis, thereby disrupting glucose homeostasis [13,46]. Therefore, twin pregnancies exposed to high levels of O_3_ are more likely to develop GDM.

Though a number of studies have assessed the trimester-specific relationship between O_3_ exposure and GDM, up to now, only one study has investigated the week-specific effects of O_3_ on the risk of GDM, indicating that the 5th to 10th gestational weeks might be a critical window for the effects of O_3_ exposure on GDM in singleton pregnancies [18]. Our findings similarly highlighted the importance of early pregnancy in twin pregnancies. In addition, we found that pre-pregnancy and mid-pregnancy were also potentially sensitive windows. Overall, it appeared that O_3_ exposure during peri-conception period and the second trimester was associated with the risk of GDM in twin pregnancies. According to the previous research, the underlying explanation might be that during these periods, developing embryos and fetuses are especially sensitive to environmental factors [16]_._ In the peri-conception period, oocyte and sperm are highly vulnerable to oxidative stress, and the demethylation of imprinted genes is crucial during the process of gamete to germ cell formation [47]. The high exposure to O_3_ leading to oxidative damage could result in the DNA methylation of the imprinted gene Igf2, which then might inhibit its gene expression [16]. Igf2 is an imprinted gene in humans that plays a key role in the development of diabetes and the inhibition of Igf2, and it has been found to be involved in maternal hyperglycemia [16]. In the second trimester, the increased production of placental hormones, such as human placental lactogen and progesterone, leads to heightened insulin resistance [48]. Additionally, weight gain and fat accumulation during mid-pregnancy could further exacerbate insulin resistance and increase the risk of GDM [49].

It is worth noting that, based on the trimester-scale analysis, we observe the significant association between O_3_ exposure and the risk of GDM during the 12 weeks before pregnancy, rather than in the first or the second trimester. In contrast, based on the week-scale analysis, we found that O_3_ exposure during specific gestational weeks both in the first and second trimesters was associated with the risk of GDM. The trimester-scale analysis adopted by our study aggregates exposure over a broad period, potentially masking short-term variations in O_3_ exposure that may have effects. By contrast, the week-scale analysis is capable of capturing finer temporal details, allowing the identification of specific weeks which might be particularly susceptible to environmental stressors like O_3_. In addition, there was evidence that the 12 weeks before pregnancy might be a critical period for the establishment of baseline insulin sensitivity, thereby having a lasting impact on glucose metabolism during pregnancy [34,50,51].

Furthermore, we explored the multiplicative and additive interaction between high O_3_ exposure (≥100 μg/m^3^) during different periods and maternal characteristics on GDM. The significant synergistic effects were observed between high O_3_ exposure during the preconception period and advanced maternal age and having history of preterm birth/abortion/stillbirth. Previous studies have shown that advanced maternal age, a history of abortion, preterm birth, and stillbirth could increase the GDM risk [52,53]. Our findings suggested that high O_3_ exposure may amplify the adverse effects of these particular maternal risk factors on the development of GDM, particularly when high exposure occurs before conception. The observed synergistic effects underscored the importance of considering both environmental and maternal characteristics in assessing GDM risk. In addition to O_3_ exposure and twin pregnancies, maternal GDM risk factors such as advanced maternal age, and a history of preterm birth, abortion, or stillbirth have been linked to higher levels of oxidative stress [54]. As previously described, oxidative stress has been associated with the risk of GDM. Thus, it is plausible that the interactions between high O_3_ exposure and maternal risk factors among twin pregnancies might be mediated through oxidative stress.

Finally, to assess the robustness of our results, we conducted double-pollutant models and sensitivity analyses in four subgroups after excluding participants of minority ethnicities, those carrying DCDA, subjects exposed to tobacco/alcohol, or those with pre-pregnancy obesity, similar results were observed in general, which further strengthen the association between O_3_ exposure during periconception periods and the risk of GDM among twin pregnant women.

Our study has several strengths. First, while the existing research predominantly addresses the effects of environmental exposures on singleton pregnancies, our study uniquely focuses on twin pregnancies, a population with distinct physiological and clinical characteristics. Notably, to our knowledge, this is the first study exploring the association between O_3_ exposure and the risk of GDM among twin pregnant women. This focus allows us to fill a critical gap in understanding how O_3_ exposure may differentially impact maternal health in this high-risk group. Second, our study identified two key exposure windows for O_3_: the 3rd week before gestation to the 2nd gestational week and from the 17th to the 19th gestational week. These findings provide novel insights into the timing of O_3_ exposure in twin pregnancies that is associated with GDM risk, offering actionable implications for intervention strategies. Third, the Cox proportion model with DLNMs was applied to identify the week-scale critical susceptible period for the effect of O_3_ exposure on GDM. The Cox proportion model with DLNMs allowed us to account for the timing of GDM diagnosis during pregnancy, providing a more detailed and precise understanding of the relationship between O_3_ exposure and GDM risk over time. Fourth, our findings provided the first evidence that high O_3_ exposure could amplify the detrimental effects of certain maternal factors on the risk of GDM.

However, our study has several limitations. First, the single-center design of the present study might raise the possibility of selection bias. Future studies with a multicenter design are warranted. Second, our findings are based on data from Shanghai, which shows relatively high ambient O_3_ levels [55,56]. However, as the most developed metropolis, Shanghai has an advanced healthcare system with widespread access to prenatal care and routine screening for GDM. These unique environmental and healthcare characteristics may influence the generalizability of our results. The critical windows for O_3_ should be examined in other regions since, in theory, exposure to different levels of O_3_ may result in different health effects. Third, although major confounders concerning the association between O_3_ exposure and the risk of GDM were considered in our analysis, other important factors, such as energy intake, were not taken into account. Fourth, the O_3_ exposure was estimated by matching the longitude and latitude of home address data, while O_3_ exposure in workplaces may introduce interferences, which potentially reduced the accuracy of exposure level measurements. Fifth, although outdoor O_3_ exposure is the primary contributor to overall exposure, which is typically used in epidemiological studies, indoor O_3_ levels may also modulate individual exposure levels, which may affect the validity of the results.

## 5. Conclusions

Our research is the first to provide evidence supporting a positive association between average O_3_ exposure during the 12 weeks before pregnancy and the development of GDM in twin pregnancies. Furthermore, our findings suggested that the 3rd week before gestation to the 2nd gestational week and the 17th to the 19th gestational week could be critical windows for higher O_3_ exposure in its relation to increased risk of GDM. Remarkably, high O_3_ exposure may amplify the adverse effects of certain maternal GDM risk factors, including advanced maternal age, and a history of preterm birth/abortion/stillbirth, particularly when high exposure occurs before conception. These findings emphasize the need for targeted public health strategies to reduce O_3_ exposure, particularly during critical periods before and during pregnancy, to mitigate the risk of GDM in twin pregnancies. Moreover, our study highlights the importance of integrating environmental factors into maternal health risk assessments, especially for high-risk populations such as those with multiple gestations.

## Figures and Tables

**Table 1:** Baseline characteristics of participants by GDM.

Characteristic	Total (n = 428)	Non-GDM (n = 356)	GDM (n = 72)	*p*
Age (years), mean ± SD	32.23 ± 3.80	32.05 ± 3.82	33.07 ± 3.60	0.038 *
Age (years), n (%)				
<35	320 (74.8)	272 (76.4)	48 (66.7)	0.113
≥35	108 (25.2)	84 (23.6)	24 (33.3)	
Ethnicity, n (%)				
Han	415 (97.0)	344 (96.6)	71 (98.6)	0.605
Other	13 (3.0)	12 (3.4)	1 (1.4)	
Education, n (%)				
Junior school or below	15 (3.5)	10 (2.8)	5 (6.9)	0.070
High school	31 (7.2)	29 (8.1)	2 (2.8)	
College or graduate school or above	382 (89.3)	317 (89.0)	65 (90.3)	
Family monthly income per capita (RMB), n (%)				
<5000	42 (9.8)	36 (10.1)	6 (8.3)	0.898
5000–7999	327 (76.4)	271 (76.1)	56 (77.8)	
≥8000	59 (13.8)	49 (13.8)	10 (13.9)	
First gestation, n (%)				
No	245 (57.2)	207 (58.1)	38 (52.8)	0.478
Yes	183 (42.8)	149 (41.9)	34 (47.2)	
Primipara, n (%)				
No	58 (13.6)	50 (14.0)	8 (11.1)	0.635
Yes	370 (86.4)	306 (86.0)	64 (88.9)	
History of abortion/preterm birth/still birth, n (%)				
No	268 (62.6)	227 (63.8)	41 (56.9)	0.338
Yes	160 (37.4)	129 (36.2)	31 (43.1)	
Chorionicity, n (%)				
MCDA/MCMA	308 (72.0)	253 (71.1)	55 (76.4)	0.440
DCDA	120 (28.0)	103 (28.9)	17 (23.6)	
Pregnancy via ART, n (%)				
No	155 (36.2)	134 (37.6)	21 (29.2)	0.219
Yes	273 (63.8)	222 (62.4)	51 (70.8)	
Preconception tobacco/alcohol use, n (%)				
No	384 (89.7)	317 (89.0)	67 (93.1)	0.418
Yes	44 (10.3)	39 (11.0)	5 (6.9)	
Pre-pregnancy BMI (kg/m^2^), mean ± SD	21.53 ± 2.88	21.37 ± 2.75	22.33 ± 3.36	0.010 *
Pre-pregnancy overweight/obesity, n (%)				0.086
No	359 (83.9)	304 (85.4)	55 (76.4)	
Yes	69 (16.1)	52 (14.6)	17 (23.6)	
Anemia, n (%)				
No	338 (79.0)	276 (77.5)	62 (86.1)	0.141
Yes	90 (21.0)	80 (22.5)	10 (13.9)	
Gestational hypertension, n (%)				
No	392 (91.6)	325 (91.3)	67 (93.1)	0.796
Yes	36 (8.4)	31 (8.7)	5 (6.9)	
Thyroid disease, n (%)				
No	367 (85.7)	307 (86.2)	60 (83.3)	0.647
Yes	61 (14.3)	49 (13.8)	12 (16.7)	

GDM: gestational diabetes mellitus; ART: assisted reproductive technology; MCMA: monochorionic monoamniotic; MCDA: monochorionic diamniotic.; DCDA: dichorionic diamniotic; BMI: Body mass index; *: *p* < 0.05 was considered statistically significant.

**Table 2:** The effects of single-pollutant and double-pollutant O_3_ exposure on GDM risk during different periods.

	Crude HR (95% CI)	Model I HR (95% CI)	Model II HR (95% CI)	Model III HR (95% CI)
Single pollution				
12 weeks before pregnancy				
Average O_3_ exposure (continuous) ^a^	1.16 (1.03, 1.31) *	1.24 (1.05, 1.45) *	1.26 (1.07, 1.48) *	1.26 (1.07, 1.48) *
Average O_3_ exposure (category)				
<100 μg/m^3^	1	1	1	1
≥100 μg/m^3^	1.91 (1.15, 3.18) *	2.83 (1.35, 5.90) *	2.75 (1.30, 5.80) *	2.89 (1.36, 6.12) *
First trimester				
Average O_3_ exposure (continuous) ^a^	1.05 (0.94, 1.17)	0.90 (0.76, 1.08)	0.88 (0.74, 1.06)	0.87 (0.73, 1.04)
Average O_3_ exposure (category)				
<100 μg/m^3^	1	1	1	1
≥100 μg/m^3^	1.25 (0.78, 2.02)	0.73 (0.37, 1.42)	0.67 (0.33, 1.35)	0.64 (0.31, 1.31)
Second trimester				
Average O_3_ exposure (continuous) ^a^	0.97 (0.86, 1.08)	1.09 (0.90, 1.33)	1.12 (0.91, 1.37)	1.11 (0.91, 1.36)
Average O_3_ exposure (category)				
<100 μg/m^3^	1	1	1	1
≥100 μg/m^3^	0.78 (0.49, 1.24)	1.15 (0.54, 2.44)	1.10 (0.50, 2.40)	1.13 (0.52, 2.47)
Double-pollution				
O_3_ + PM_2.5_				
12 weeks before pregnancy				
Average O_3_ exposure (continuous) ^a^	1.27 (1.09, 1.48) **	1.26 (1.07, 1.49) **	1.28 (1.09, 1.52) **	1.29 (1.09, 1.53) **
Average O_3_ exposure (category)				
<100 μg/m^3^	1	1	1	1
≥100 μg/m^3^	2.99 (1.48, 6.03) **	3.06 (1.44, 6.52) **	3.00 (1.39, 6.47) **	3.19 (1.46, 6.98) **
First trimester				
Average O_3_ exposure (continuous) ^a^	0.98 (0.84, 1.15)	0.91 (0.76, 1.09)	0.89 (0.74, 1.07)	0.87 (0.73, 1.05)
Average O_3_ exposure (category)				
<100 μg/m^3^	1	1	1	1
≥100 μg/m^3^	0.99 (0.53, 1.85)	0.73 (0.37, 1.45)	0.69 (0.34, 1.40)	0.65 (0.31, 1.32)
Second trimester				
Average O_3_ exposure (continuous) ^a^	0.91 (0.78, 1.07)	1.09 (0.89, 1.33)	1.12 (0.91, 1.37)	1.11 (0.91, 1.36)
Average O_3_ exposure (category)				
<100 μg/m^3^	1	1	1	1
≥100 μg/m^3^	0.59 (0.32, 1.09)	1.15 (0.54, 2.44)	1.10 (0.50, 2.41)	1.15 (0.53, 2.50)
O_3_ + PM_10_				
12 weeks before pregnancy				
Average O_3_ exposure (continuous) ^a^	1.18 (1.05, 1.34) **	1.22 (1.04, 1.44) *	1.24 (1.04, 1.46) *	1.23 (1.03, 1.45) *
Average O_3_ exposure (category)				
<100 μg/m^3^	1	1	1	1
≥100 μg/m^3^	2.17 (1.27, 3.68) **	2.79 (1.30, 5.96) **	2.66 (1.24, 5.73) **	2.69 (1.24, 5.82) *
First trimester				
Average O_3_ exposure (continuous) ^a^	0.99 (0.86, 1.13)	0.92 (0.77, 1.10)	0.90 (0.75, 1.09)	0.89 (0.73, 1.07)
Average O_3_ exposure (category)				
<100 μg/m^3^	1	1	1	1
≥100 μg/m^3^	0.97 (0.56, 1.68)	0.76 (0.38, 1.52)	0.72 (0.35, 1.50)	0.68 (0.33, 1.41)
Second trimester				
Average O_3_ exposure (continuous) ^a^	0.97 (0.84, 1.12)	1.12 (0.91, 1.38)	1.15 (0.93, 1.43)	1.15 (0.93, 1.43)
Average O_3_ exposure (category)				
<100 μg/m^3^	1	1	1	1
≥100 μg/m^3^	0.74 (0.41, 1.33)	1.20 (0.55, 2.62)	1.15 (0.50, 2.60)	1.25 (0.55, 2.84)
O_3_ + SO_2_				
12 weeks before pregnancy				
Average O_3_ exposure (continuous) ^a^	1.17 (1.03, 1.32) *	1.24 (1.05, 1.45) **	1.26 (1.07, 1.48) **	1.26 (1.07, 1.48) **
Average O_3_ exposure (category)				
<100 μg/m^3^	1	1	1	1
≥100 μg/m^3^	1.95 (1.17, 3.26) *	2.90 (1.38, 6.11) **	2.80 (1.32, 5.93) **	2.91 (1.37, 6.19) **
First trimester				
Average O_3_ exposure (continuous) ^a^	1.03 (0.91, 1.17)	0.90 (0.75, 1.08)	0.88 (0.73, 1.06)	0.87 (0.72, 1.05)
Average O_3_ exposure (category)				
<100 μg/m^3^	1	1	1	1
≥100 μg/m^3^	1.15 (0.69, 1.95)	0.71 (0.35, 1.42)	0.66 (0.32, 1.37)	0.63 (0.30, 1.31)
Second trimester				
Average O_3_ exposure (continuous) ^a^	1.00 (0.87, 1.16)	1.10 (0.90, 1.35)	1.12 (0.91, 1.38)	1.11 (0.91, 1.37)
Average O_3_ exposure (category)				
<100 μg/m^3^	1	1	1	1
≥100 μg/m^3^	0.85 (0.48, 1.50)	1.15 (0.54, 2.46)	1.09 (0.49, 2.41)	1.13 (0.52, 2.47)
O_3_ + NO_2_				
12 weeks before pregnancy				
Average O_3_ exposure (continuous) ^a^	1.28 (1.11, 1.48) ***	1.26 (1.08, 1.48) **	1.28 (1.09, 1.51) **	1.29 (1.09, 1.52) **
Average O_3_ exposure (category)				
<100 μg/m^3^	1	1	1	1
≥100 μg/m^3^	3.36 (1.68, 6.74) ***	3.31 (1.55, 7.05) **	3.30 (1.52, 7.16) **	3.46 (1.58, 7.57) **
First trimester				
Average O_3_ exposure (continuous) ^a^	0.97 (0.83, 1.14)	0.91 (0.76, 1.08)	0.89 (0.74, 1.07)	0.88 (0.73, 1.05)
Average O_3_ exposure (category)				
<100 μg/m^3^	1	1	1	1
≥100 μg/m^3^	0.95 (0.51, 1.76)	0.74 (0.37, 1.46)	0.69 (0.34, 1.41)	0.65 (0.32, 1.34)
Second trimester				
Average O_3_ exposure (continuous) ^a^	1.06 (0.90, 1.25)	1.10 (0.90, 1.34)	1.13 (0.92, 1.39)	1.12 (0.91, 1.37)
Average O_3_ exposure (category)				
<100 μg/m^3^	1	1	1	1
≥100 μg/m^3^	0.97 (0.49, 1.92)	1.17 (0.55, 2.49)	1.11 (0.50, 2.44)	1.14 (0.52, 2.50)
O_3_ + CO				
12 weeks before pregnancy				
Average O_3_ exposure (continuous) ^a^	1.19 (1.05, 1.36) **	1.24 (1.05, 1.45) **	1.25 (1.06, 1.48) **	1.25 (1.06, 1.48) **
Average O_3_ exposure (category)				
<100 μg/m^3^	1	1	1	1
≥100 μg/m^3^	2.36 (1.30, 4.29) **	2.94 (1.39, 6.20) **	2.87 (1.35, 6.13) **	2.97 (1.39, 6.37) **
First trimester				
Average O_3_ exposure (continuous) ^a^	1.04 (0.92, 1.18)	0.87 (0.72, 1.05)	0.86 (0.70, 1.04)	0.84 (0.69, 1.02)
Average O_3_ exposure (category)				
<100 μg/m^3^	1	1	1	1
≥100 μg/m^3^	1.20 (0.71, 2.05)	0.64 (0.31, 1.33)	0.60 (0.28, 1.29)	0.56 (0.26, 1.23)
Second trimester				
Average O_3_ exposure (continuous) ^a^	0.96 (0.84, 1.10)	1.10 (0.90, 1.34)	1.12 (0.91, 1.38)	1.11 (0.91, 1.36)
Average O_3_ exposure (category)				
<100 μg/m^3^	1	1	1	1
≥100 μg/m^3^	0.73 (0.43, 1.25)	1.14 (0.53, 2.42)	1.08 (0.49, 2.38)	1.13 (0.52, 2.46)

O_3_: ozone; PM_2.5_: particulate matter 2.5; PM_10_: particulate matter 10; SO_2_: sulfur dioxide; NO_2_: nitrogen dioxide; CO: carbon monoxide; GDM: gestational diabetes mellitus; HR: hazard ratio; CI: confidence interval; ^a^: risk of GDM with an increase of 10 μg/m^3^ in O_3_ concentration; *: *p* < 0.05; **: *p* < 0.01; ***: *p* < 0.001; Model 1 adjusted for temperature and relative humidity. Model II adjusted for those factors included in model I and sociodemographic characteristics (advanced maternal age, ethnicity, education level, and family income), history of preterm birth/abortion/still birth, first gestation, primipara, chorionicity, and pregnancy via assisted reproductive technology. Model III adjusted for those factors included in model II and living habits before pregnancy (maternal tobacco/alcohol use, pre-pregnancy body mass index) and physical conditions (anemia, thyroid disease, and gestational hypertension).

## Data Availability

The data presented in this study are available on request from the corresponding author due to the privacy.

## Supplementary Materials

The following supporting information can be downloaded at: https://www.mdpi.com/article/10.3390/toxics13020117/s1, Figure S1: Flowchart of study population selection; Figure S2: Levels of O_3_ by Month in Shanghai from 2018 to 2023; Table S1: Levels of Air Pollutant Concentrations during Different Periods in the STPBC Study; Figure S3: Risk of GDM with Average O_3_ Exposure by Month before Pregnancy; Table S2: Characteristics of Participants by High O_3_ Exposure during 12 weeks before Pregnancy; Table S3: Interaction between high O_3_ exposure (≥100 μg/m^3^) during different trimesters and maternal characteristics on GDM risks; Table S4: Sensitivity analysis 1: HRs of GDM associated with average O_3_ exposure in 12 weeks before pregnancy; Figure S4: Sensitive analyses 2: Weekly associations between O_3_ exposure during 12 weeks before pregnancy and GDM in different population and models.
