# Supplementary material for: Ozone Exposure and Gestational Diabetes in Twin Pregnancies: Exploring Critical Windows and Synergistic Risks
Source: Toxics. 2025 Feb 1;13(2):117. doi: 10.3390/toxics13020117 (PMC11860467; doi:10.3390/toxics13020117)
Supplement: Supplementary file 1 [file toxics-13-00117-s001.zip › toxics-3406864-supplementary.pdf]

Ozone Exposure and Gestational Diabetes in Twin Pregnancies: Exploring Critical Windows and Synergistic Risks

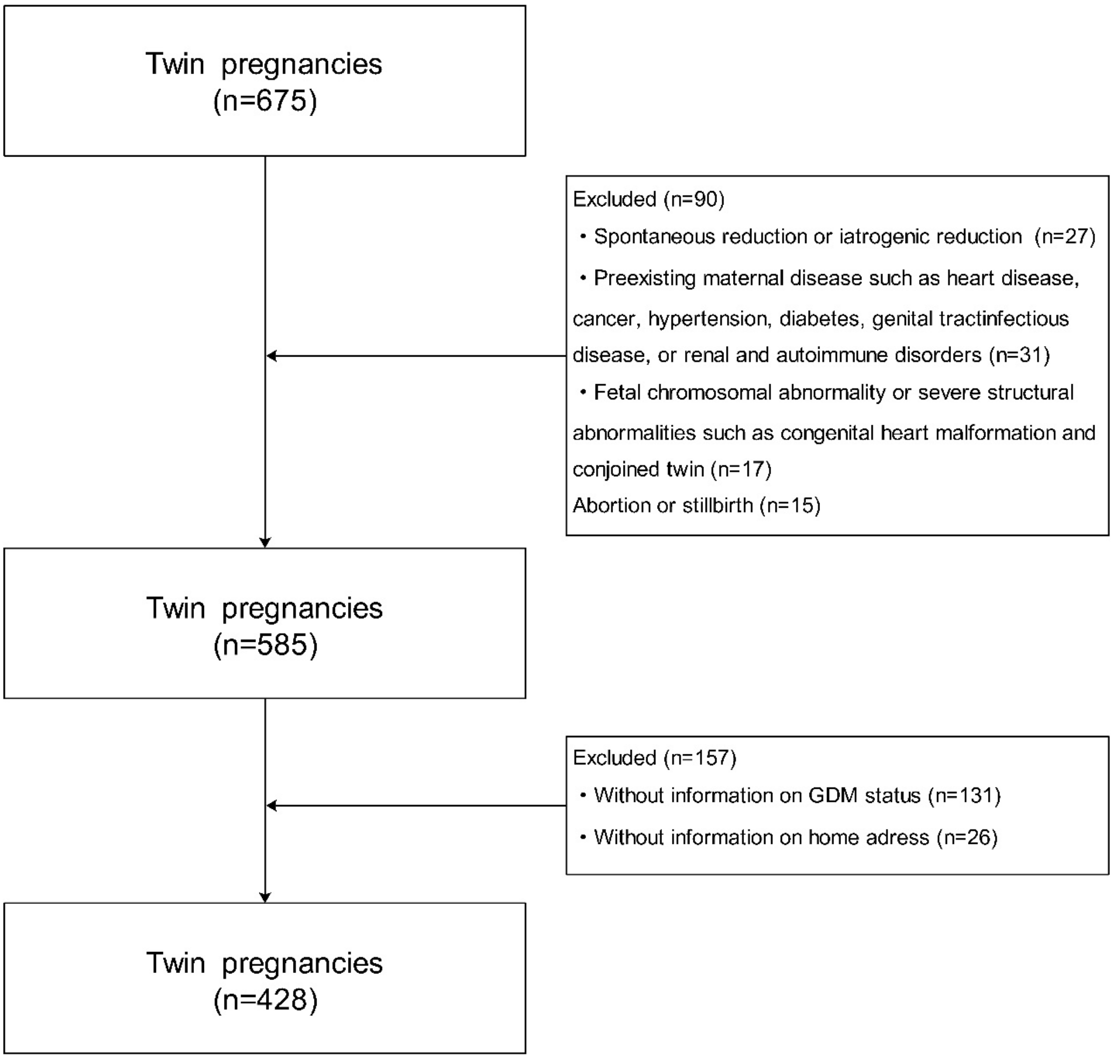

Figure S1. Flowchart of study population selection

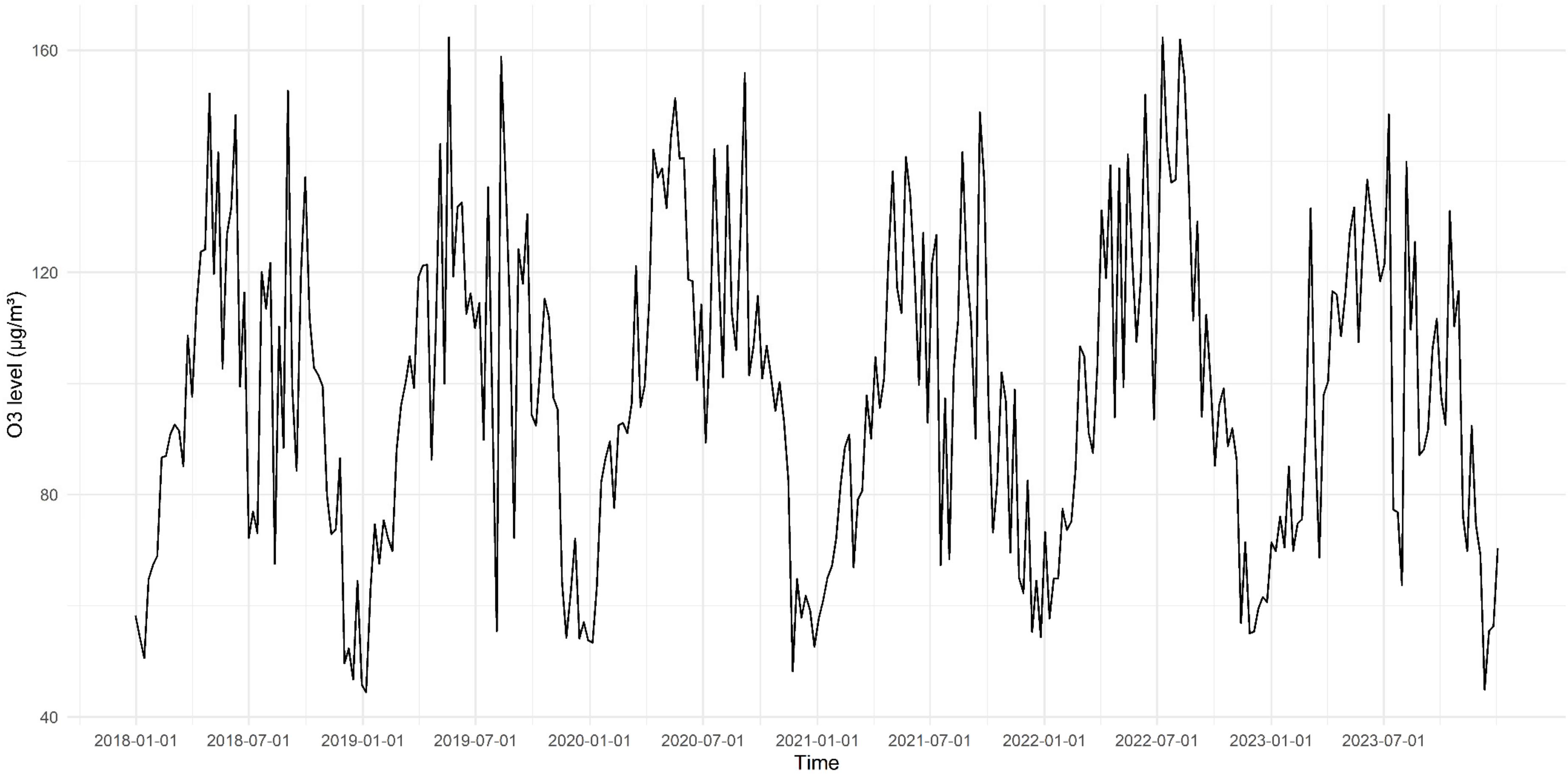

Figure S2. Levels of O_3 by month in Shanghai from 2018 to 2023

Table S1. Levels of air pollutant concentrations during different periods in the STPBC study

Period	Pollutant	Min.	25th	50th	75th	Max.	IQR
**12 weeks before pregnancy**	O_3 (μg/m^3)	52.27	80.79	104.87	116.43	133.25	35.64
	$PM_{2.5}$ (μg/m^3)	13.56	25.23	30.70	39.24	55.12	14.01
	PM_{10} (μg/m^3)	29.94	39.46	47.23	54.70	70.19	15.24
	SO_2 (μg/m^3)	4.37	6.21	7.10	7.92	13.69	1.71
	NO_2 (μg/m^3)	23.68	33.75	38.60	48.43	59.58	14.68
	CO (mg/m^3)	0.47	0.60	0.64	0.70	0.88	0.10
**First trimester**	O_3 (μg/m^3)	58.94	79.68	105.89	115.48	136.70	35.80
	$PM_{2.5}$ (μg/m^3)	13.25	24.49	30.01	38.16	52.80	13.67
	PM_{10} (μg/m^3)	28.07	37.49	45.07	54.19	70.7	16.70
	SO_2 (μg/m^3)	4.32	5.62	6.59	7.41	12.35	1.79
	NO_2 (μg/m^3)	21.56	31.75	37.99	47.62	57.13	15.87
	CO (mg/m^3)	0.48	0.59	0.64	0.70	0.80	0.11
**Second trimester**	O_3 (μg/m^3)	55.57	80.46	103.24	117.58	140.76	37.12
	$PM_{2.5}$ (μg/m^3)	15.72	24.86	31.00	37.38	53.01	12.52
	PM_{10} (μg/m^3)	27.92	39.05	46.35	53.55	60.47	14.50
	SO_2 (μg/m^3)	3.88	5.41	6.30	7.26	8.62	1.85
	NO_2 (μg/m^3)	20.12	33.06	39.59	47.52	56.82	14.46
	CO (mg/m^3)	0.48	0.61	0.66	0.70	0.79	0.09

STPBC: Shanghai Twin Pregnancy Birth Cohort; IQR: interquartile range

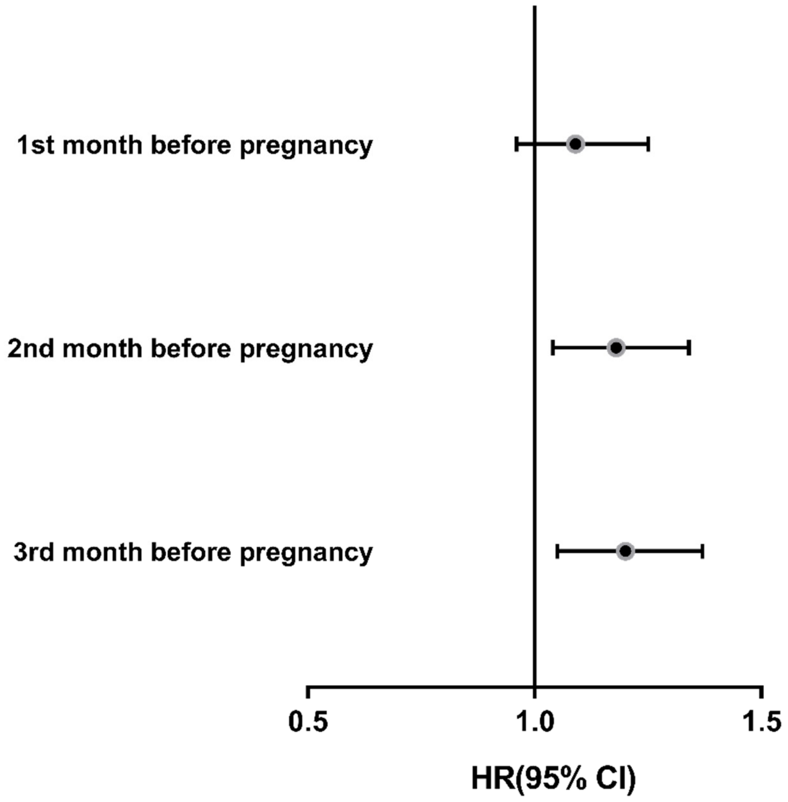

Figure S3. Risk of GDM with average O_3 exposure by month before pregnancy

GDM: gestational diabetes mellitus; risk of GDM with an increase of 10 μg/m^3 in O_3 concentration; adjusted for temperature and relative humidity, sociodemographic characteristics (advanced maternal age, ethnicity, education level, family income), history of preterm birth/abortion/still birth, first gestation, primipara, chorionicity, pregnancy via assisted reproductive technology, pregnancy health indicators, including maternal tobacco/alcohol use, pre-pregnancy body mass index, anemia, thyroid disease, and gestational hypertension.

Table S2. Characteristics of participants by high O_3 exposure during 12 weeks before pregnancy

	**Total (n=428)**	**Low exposure[a] (n=184)**	**High exposure[b] (n=244)**	***P***
Age(years), mean±SD	32.23±3.80	32.23±3.68	32.22±3.89	0.97
Age(years), n (%)				0.81
<35	320 (74.8)	136 (73.9)	184 (75.4)	
≥35	108 (25.2)	48 (26.1)	60 (24.6)	
BMI(kg/m^2), mean±SD	21.53±2.88	21.80±3.28	21.33±2.53	0.10
Pre-pregnancy obesity, n (%)				0.44
No	418 (97.7)	149 (96.7)	210 (98.4)	
Yes	10 (2.3)	35 (3.3)	34 (1.6)	

[a]: average O_3 exposure during 12 weeks before pregnancy <100 μg/m^3; **[b]:** average O_3 exposure during 12 weeks before pregnancy ⩾100 μg/m^3; BMI: body mass index.

Table S3. Interaction between high O_3 exposure (⩾100 μg/m^3) during different trimesters and maternal characteristics on GDM risks

	P for MI	RERI (95% CI)
**First trimester**		
Advanced maternal age	0.300	0.40 (-1.24, 1.61)
History of abortion/PB/SB	0.420	-0.32 (-4.55, 1.21)
**Second trimester**		
Advanced maternal age	0.367	-0.27 (-4.15, 2.60)
History of abortion/PB/SB	0.126	0.56 (-1.07, 2.10)

GDM: gestational diabetes mellitus; MI: multiplicative interaction; RERI: Relative excess risk due to interaction; PB: preterm birth; SB: still birth. Adjusted for temperature and relative humidity, sociodemographic characteristics (advanced maternal age, ethnicity, education level, family income), history of preterm birth/abortion/still birth, first gestation, primipara, chorionicity, pregnancy via assisted reproductive technology, pregnancy health indicators, including maternal tobacco/alcohol use, pre-pregnancy body mass index, anemia, thyroid disease, and gestational hypertension.

Table S4. Sensitivity analysis 1: HRs of GDM associated with average O_3 exposure in 12 weeks before pregnancy

	Other ethnicities excluded	DCDA excluded	Tobacco/alcohol use excluded	Pre-pregnancy obesity excluded
**12 weeks before pregnancy**				
Average O_3 level (continuous)[a]	1.28(1.09,1.51)**	1.22(1.01,1.49)**	1.23(1.04,1.46)*	1.26(1.07,1.49)**
Average O_3 level (category)				
<100 μg/m^3	1	1	1	1
≥100 μg/m^3	2.86(1.35,6.07)**	3.42(1.38, 8.44)*	2.45(1.13,5.29)*	2.85(1.34,6.08)**

GDM: gestational diabetes mellitus; DCDA: dichorionic diamniotic; [a]: risk of GDM with an increase of 10 μg/m^3 in O_3 concentration; *:$P<0.05$; **:$P<0.01$; Adjusted for temperature and relative humidity, sociodemographic characteristics (advanced maternal age, ethnicity, education level, family income), history of preterm birth/abortion/still birth, first gestation, primipara, chorionicity, pregnancy via assisted reproductive technology, pregnancy health indicators, including maternal tobacco/alcohol use, pre-pregnancy body mass index, anemia, thyroid disease, and gestational hypertension.

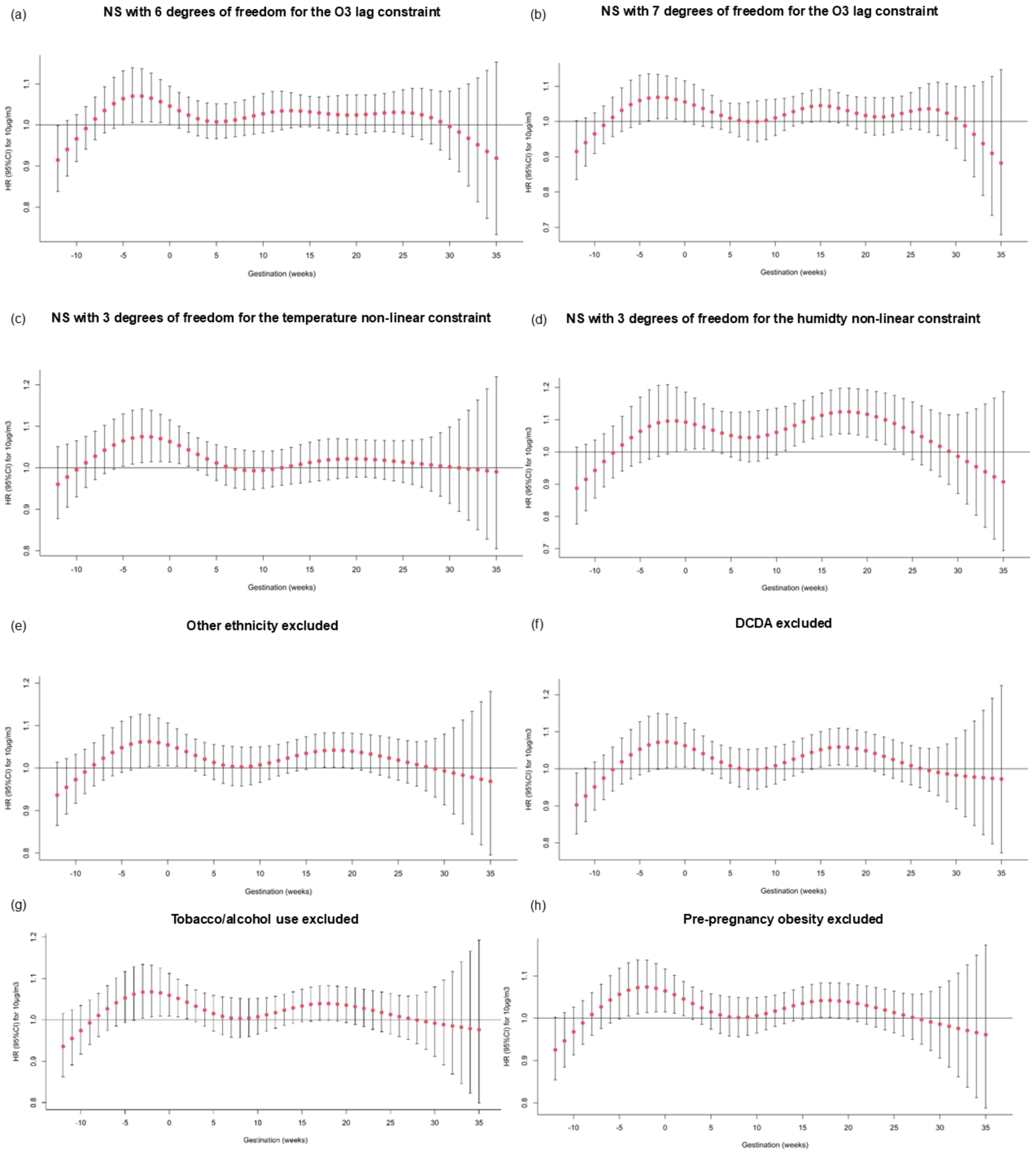

Figure S4. Sensitive Analyses 2: Weekly Associations between O_3 Exposure during 12 Weeks before Pregnancy and GDM in Different Population and Models**.**

NS: natural splines; DCDA: dichorionic diamniotic; Adjusted for temperature and relative humidity, sociodemographic characteristics (advanced maternal age, ethnicity, education level, family income), history of preterm birth/abortion/still birth, first gestation, primipara, chorionicity, pregnancy via assisted reproductive technology, pregnancy health indicators, including maternal tobacco/alcohol use, pre-pregnancy body mass index, anemia, thyroid disease, and gestational hypertension.
